# Self-Toughening and Self-Enhancement Poly(arylene ether nitrile) with Low Dielectric Constant by Solid Crosslinking Reaction

**DOI:** 10.3390/polym11091403

**Published:** 2019-08-27

**Authors:** Lifen Tong, Xiting Lei, Guangyao Yang, Xiaobo Liu

**Affiliations:** Research Branch of Advanced Functional Materials, School of Materials and Energy, University of Electronic Science and Technology of China, Chengdu 610054, China

**Keywords:** low dielectric constant, poly(arylene ether nitrile), thermal properties, self-toughening, self-enhancement

## Abstract

A novel poly(arylene ether nitrile) terminated with hydroxyl groups (PEN–OH) was synthesized successfully. The effects of heat-treatment temperature on the thermal properties, mechanical properties, and dielectric properties of the PEN–OH films were studied in detail. Due to the cross-linking reaction occurring, at high temperature, among the nitrile groups on the side of the PEN–OH main chain to form a structurally stable triazine ring, the structure of materials changes from a linear structure to a bulk structure. Thus, the thermal properties and mechanical properties were improved. In addition, the occurrence of cross-linking reactions can reduce the polar groups in the material, leading to the decrease of dielectric constant. As the heat-treatment temperature increased, the glass-transition temperature increased from 180.6 °C to 203.6 °C, and the dielectric constant decreased from 3.4 to 2.8 at 1 MHz. Proper temperature heat-treatment could improve the tensile strength, as well as the elongation, at the break of the PEN–OH films. Moreover, because of the excellent adhesive property of PEN–OH to copper foil, a double-layer flexible copper clad laminate (FCCL) without any adhesives based on PEN–OH was prepared by a simple hot-press method, which possessed high peel strength with 1.01 N/mm. Therefore, the PEN–OH has potential applications in the electronic field.

## 1. Introduction

With the rapid development of consumer electronics, flexible copper clad laminate (FCCL) with high temperature, high frequency, and high transmission speed has attracted extensive research interest in the academic and commercial fields. Matrix resin is an important component of the FCCL, and the adhesion property of the matrix resin to the copper is a significant factor in the FCCL. Poly(arylene ether nitrile) (PEN) has received extensive attention due to its excellent heat resistance, corrosion resistance, high strength, and high modulus [1,2,3,4,5,6,7,8]. Due to the presence of the nitrile groups, PEN has stronger polarizability than that of other poly(arylene ether)s, which appears to promote the adhesion of the polymers to a variety of substrates [9]. In present work, the presence of the nitrile groups makes the PEN have good adhesion to copper, so a double-layer flexible copper clad laminate can be prepared by a simple hot pressing method without binder, which will greatly reduce the dielectric loss in signal transmission and improve the thermal properties of the FCCL [10,11,12]. Therefore, the PEN will provide great possibilities for flexible, high temperature resistance, high frequency and high transmission speed copper clad laminate materials.

In order to achieve high-speed transmission of signals, matrix resin for copper clad laminates is required to have a low dielectric constant and low dielectric loss. Conventional poly(arylene ether nitrile) shows a dielectric constant of about 4.0 [5,13,14]. According to previous reports, the introduction of fluorine can greatly reduce the dielectric constant of the polymers [15,16,17,18,19]. Fluorinated poly(norborneneimide) prepared by Fujiwara [16], which contains a large fluorinated aryl group in the side chain, has a dielectric constant of 2.31. Patrick [20] designed and synthesized a high-fluorinated polymer, in which the fluorine content can be adjusted. It was found that the higher the mass fraction of fluorine in the repeating unit of the polymer, the lower the dielectric constant of the polymer. The lowest dielectric constant of the polymer can be reduced to 2.17. The introduction of fluorine reduces the dielectric constant of the polymer for the following two reasons: On the one hand, the fluorine atom is the most electronegative element, leading to that the fluorocarbon bond having a small degree of polarization. On the other hand, the fluorine-containing group has a large volume, which can increase the free volume of the polymer matrix [21]. Therefore, a novel poly(arylene ether nitrile) containing fluorine element was synthesized successfully. Although the presence of the nitrile groups improves the adhesion to copper, it also increases the polarity of the molecules. Due to the cross-linking property of the nitrile groups, at high temperature, forming the structurally stable triazine rings [1,22], the polarity of the molecules decreases, leading to the decrease of the dielectric constant of the PEN.

According to the previous reports, the phenolic hydroxyl group is an effective catalyst for nitrile cross-linking reactions [15,23]. Thus, in this work, hydroxyl terminated poly(arylene ether nitrile) (PEN–OH) was prepared, and its molecular weight can be adjusted by the molar ratio of Bisphenol-AF to 2,6-dichlorobenzonitrile. Then, the effects of the different temperature treatments on thermal properties, mechanical properties, and electrical properties were investigated in detail. Finally, taking the advantage of the excellent adhesive property of PEN–OH to copper foil, a double-layer FCCL without any adhesives was prepared by a simple hot-press method. The hot-press temperature has an effect on the formation of the PEN–OH and the crosslinking degree, which will affect the adhesion between the copper foil and the resin. Thus, the effects of different hot pressing temperatures on the peel strength of FCCL were also investigated. Through this series of studies, phenolic hydroxyl terminated poly(arylene ether nitrile) containing fluorine will be an excellent candidate for high temperature, high frequency, and high speed dielectric substrate materials.

## 2. Materials and Methods

### 2.1. Materials

Bisphenol-AF(BPAF) was obtained from Aladdin Chemicals (Shanghai, China). 2,6-Dichlorobenzonitrile (DCBN), K_2_CO_3_, *N*-methyl-2-pyrrolidone (NMP), and toluene were all purchased from Chengdu Kelong Chemical Co., Ltd. (Chengdu, China). All the chemical reagents were used without further purification.

### 2.2. Synthesis of PEN–OH

**Figure 1:**
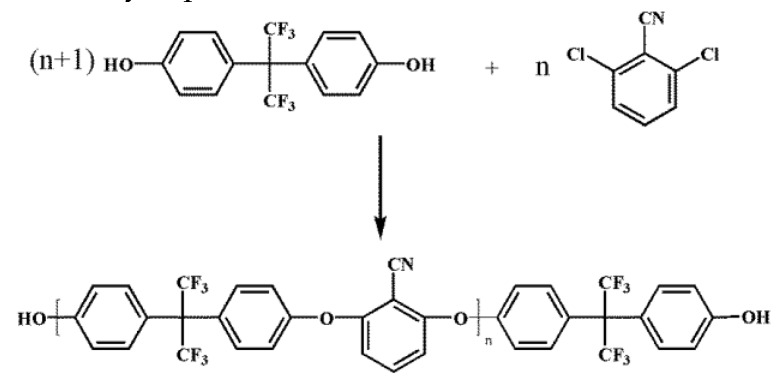
Synthetic route of hydroxyl terminated poly(arylene ether nitrile) (PEN-OH).

The PEN–OH was synthesized by nucleophilic substitution polycondensation according to the previous reports [24], and the molecular weight can be adjusted by the molar ratio of BPAF to DCBN. The detail synthetic route of PEN–OH was shown in Figure 1. In present work, we prescribed the molar ratio of BPAF to DCBN as 76:75. The theoretical value of the PEN–OH molecular weight is 3.8 × 10^4^, and the molecular weight obtained by experiment is 4.3 × 10^4^, which was obtained by GPC.

### 2.3. Preparation of PEN–OH Crosslinking Films

The PEN–OH films were prepared by simple casting method. Firstly, the PEN–OH was dissolved in the NMP to obtain a light brown solution. Then, the PEN–OH solution was cast on a clean glass plate and dried in an oven according to the procedure of 80 °C, 100 °C, 120 °C, 160 °C, and 200 °C for 1 h, respectively. Afterwards, the films were treated at 200 °C, 320 °C, 340 °C, and 360 °C for 4 h and labelled as a, b, c and d, respectively.

### 2.4. Preparation of FCCL Based on PEN–OH

The preparation of the FCCL based on PEN–OH was carried out as follows: Firstly, put the mold with the size of 100 mm in length, 50 mm in width and 0.1 mm in thickness on the copper foil. Then, the PEN–OH powders were loaded into the mold. After that, another copper foil was placed on the mold filled with PEN–OH powders. Lastly, the samples were hot-pressed at different temperatures (300 °C, 320 °C, and 340 °C) under 10 MPa for 4 h to prepare FCCL.

### 2.5. Characterization

Gel Permeation Chromatography (GPC) analysis was conducted with a PL-GPC220 system using polystyrene as standard and THF as the eluent. The FTIR spectra of the PEN–OH films were recorded on NICOLET MX-1E (Waltham, MA, USA) Fourier transform infrared spectrometer between 4000 and 400 cm^−1^.The cross-sectional morphologies of the films were observed with scanning electron microscope (SEM) (JSM-5900LV, JEOL, Tokyo, Japan) operating at 20 kV. The thermal properties of the PEN–OH films were performed on TA Instrument DSC-Q100 (New Castle, DE, USA) with a heating rate of 10 °C/min from room temperature to 300 °C under a nitrogen flow rate of 50 mL/min. Thermal gravimetric analysis of the PEN–OH films was obtained with a TA Instruments TGA-Q50 at a heating rate of 20 °C/min from room temperature to 600 °C under nitrogen atmosphere. The mechanical properties of the PEN–OH films were investigated by SANS CMT6104 Series Desktop Electromechanical Universal Testing Machine with a sample size of 10 mm × 100 mm. The average value of the five samples was obtained. Dielectric measurements were performed by using a dielectric analyzer (DEA 2970, TA Instruments). Peel strength of the FCCL was measured by a XLW (PC) intelligent electronic tensile testing machine. The test samples were 15 mm in width and 200 mm in length, respectively. The gel contents of the PEN–OH sheets, prepared by different heat-treating temperatures, were determined by the Soxhlet extraction and the NMP was employed as solvent. After the extraction, the extracted samples were vacuum-dried until the weight became constant. The gel content was calculated by the formula as follows: Gel fraction = (*W*_1_/*W*_2_) × 100%, where *W*_1_ is the dry weight of the sample after extraction, and W_2_ is the initial weight of the sample.

## 3. Results and Discussion

**Figure 2:**
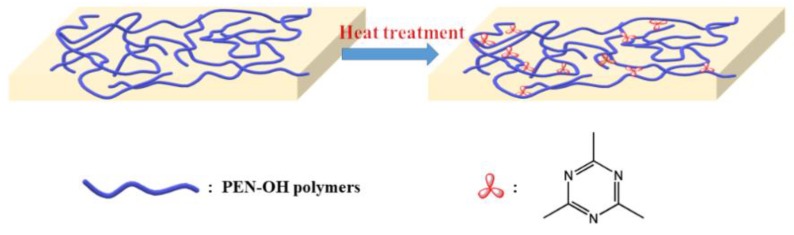
Diagram of the PEN–OH cross-linking reaction.

As shown in Figure 2, the hydroxy-terminated poly(aryl ether nitrile) has a linear structure before being heat-treated. At the presence of the catalysis of hydroxyl group, a cross-linking reaction will occur between the nitrile groups on side of the molecular chain to form a structurally stable triazine ring, which will be demonstrated later in the FTIR analysis. As the sample structure transformed from liner structure into a bulk structure, its thermal performance including the glass transition temperature and thermal decomposition temperature will be greatly improved. At the same time, its mechanical strength will also be greatly improved. These performances will be discussed in the next analysis.

**Figure 3:**
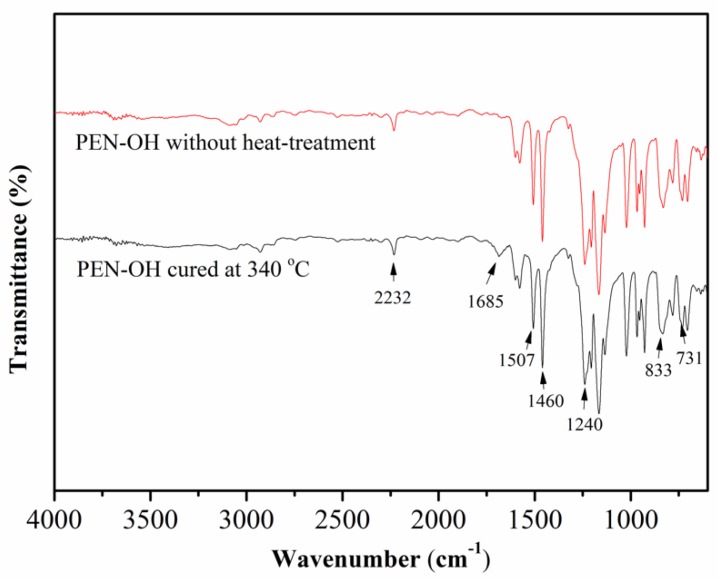
FTIR spectra of the PEN–OH before and after heat-treatment.

In order to verify the cross-linking reaction among the PEN–OH, the structures of the synthesized PEN–OH before and after heat-treatment were characterized by FTIR and the results are shown in Figure 3. As can be seen, two characteristic stretching bands emerged at 833 cm^−1^ and 731 cm^−1^ and the other two sharp and strong characteristic absorption bands at 1507 cm^−1^ and 1460 cm^−1^ were assigned to benzene rings. The absorption band emerging at 1240 cm^−1^ was clearly observed, which is attributed to skeleton vibration of the ether. These results suggest the existence of aryl ether (Ar–O–Ar) [25]. The characteristic stretching band appearing at around 2232 cm^−1^ suggested that the nitrile groups existed in the polymer [26]. Most importantly, a new characteristic absorption band appearing at 1685 cm^−1^ in PEN–OH with heat-treatment was assigned to the stretching vibration of –C=N– [27], indicating the formation of triazine ring, which provided supports for the cross-linking reactions among the PEN–OH.

**Figure 4:**
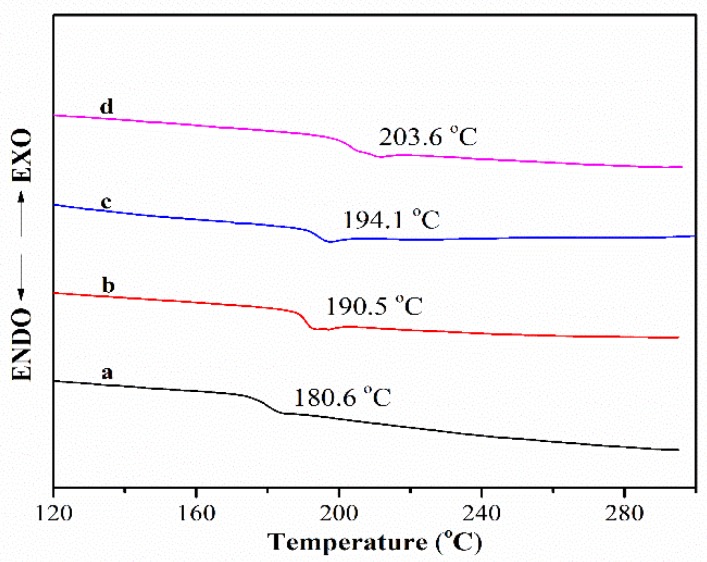
DSC of PEN–OH films heat-treated at different temperatures.

Figure 4 shows the DSC curves of the PEN–OH films with different temperature treatments. It can be seen that the glass transition temperature (*T_g_*) increases as the heat treating temperature increases. When the heat treating temperature reaches 360 °C, the *T_g_* of the cross-linked film increases to 203.6 °C, which is increased by 23.0 °C compared with that of the sample treated at 200 °C. At high temperature, the nitrile groups will react with each other to from a triazine ring, making the sample transform from a linear structure to a three-dimensional network structure at the presence of the hydroxyl groups, so that the *T_g_* increases.

**Figure 5:**
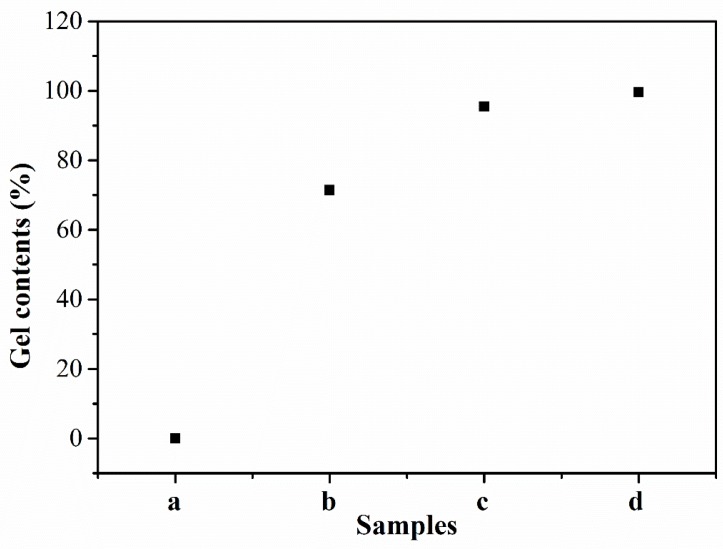
Gel contents of PEN–OH films heat-treated at different temperatures.

To further investigate the effect of different temperature treatments on the cross-linking reaction, the gel contents of the PEN–OH films, which can reflect the cross-linking degree of the polymers, were measured by Soxhlet extraction, and the results were shown in Figure 5. It can be seen that the sample treated at 200 °C was completely dissolved in the NMP with heating reflux, so the obtained gel content is 0%, indicating that this sample did not undergo a cross-linking reaction. As the treating temperature increases, the gel contents of the PEN–OH films increase gradually. When the heat-treating temperature reaches 360 °C, the gel content of the sample is up to 99.5%. The increase of the cross-linking degree will lead to the increase of the *T*_g_, which is consistent with DSC analysis results.

**Figure 6:**
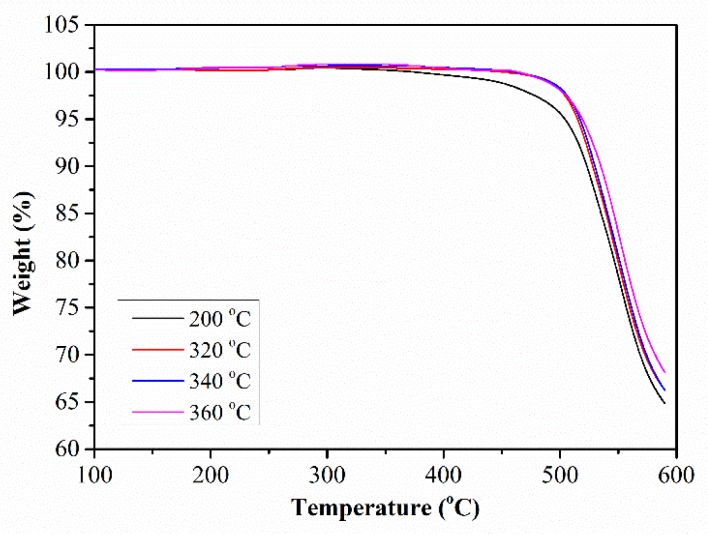
TGA of PEN–OH films heat-treated at different temperatures.

Due to the cross-linking reaction occurring at a high temperature in this PEN–OH system, the thermostability of the PEN–OH films will be improved after heat-treatment. Figure 6 shows the TGA curves of the PEN–OH films with different heat-treatment temperatures. It can be seen that all the TGA curves of the PEN–OH films show similar thermal decomposition behavior. The temperatures corresponding to the weight loss of 5 wt% (*T*_5%_) of the PEN–OH are all above 500 °C, and the temperatures corresponding to the fastest thermal decomposition are about 550 °C. It is due to the fact that these samples possess similar structures. As the heat-treatment temperature increases, *T*_5%_ of PEN–OH increases from 504.3 °C to 519.2 °C, and the char yield at the temperature of 600 °C increases from 64.8% to 68.1%. These results indicate that the thermal properties can be improved by the heat-treatment.

**Figure 7:**
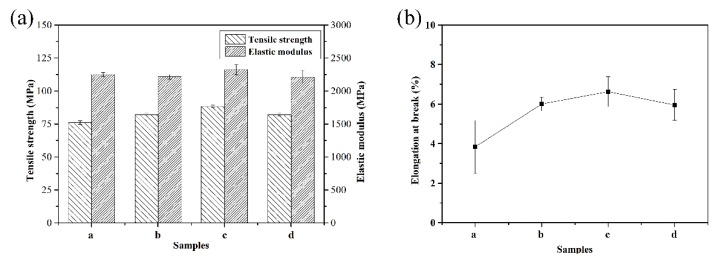
Mechanical properties of PEN–OH films. (**a**) Tensile strength and elastic modulus (**b**) elongation at the break.

Figure 7 shows the mechanical properties of the PEN–OH films with different heat-treating temperatures. It is observed from Figure 7a that, firstly, the tensile strength increases and then decreases. Sample c, which was treated at 340 °C, possesses the best tensile strength of 88.2 MPa. This was 16.1% greater than that of sample a without heat-treatment. The improvement of the tensile strength is attributed to the cross-linking reaction at the high temperature. However, there is a downward trend when the heat-treating temperature continues to rise, and the tensile strength of sample d dropped to 82.1 MPa, but it is still higher than sample a. For sample d, the decrease of the tensile strength is mainly due to its excessive degree of cross-linking, so that its brittleness increases. The tensile modulus of these films does not change much, all around 2.2 GPa. Figure 7b shows the elongation at break of the PEN–OH films with different heat-treating temperatures. The value of elongation at the break depends on the flexibility of the molecular chain of the matrix resin. In other words, the better the toughness of the molecular weight, the greater the elongation at the break. It can be seen that the elongation at the break of PEN–OH films shows a tendency to increase first and then decrease with the heat-treating temperatures increasing. Therefore, the result of the elongation at break of the PEN–OH films indicates that the toughness of the PEN–OH films can be improved through suitable temperature heat-treatment without any addition agent. Combined with the analysis of gel content (Figure 5), it draws a conclusion that proper cross-linking density not only increases tensile strength, but also increases elongation at the break.

**Figure 8:**
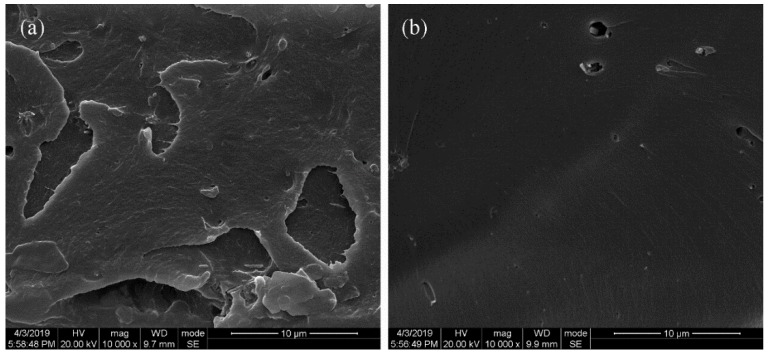
SEM of the fracture surface of PEN–OH films. (**a**) without heat-treatment (**b**) cured at 340 °C.

Figure 8 displays the SEM images of the PEN–OH films before and after heat-treatment to investigate the microscopic network structures formed by cross-linking reactions among the nitrile groups at a high temperature. As shown in Figure 8a, there is remarkable toughness fracture surface morphology in the PEN–OH film without heat-treatment. However, as demonstrated in Figure 8b, the PEN–OH film with heat-treatment exhibited a brittle fracture, which is caused by the cross-linking reaction among the nitrile groups. Therefore, the linear structure transforms to three-dimensional network structure, which is beneficial to improve the mechanical and thermal performance of the system.

**Figure 9:**
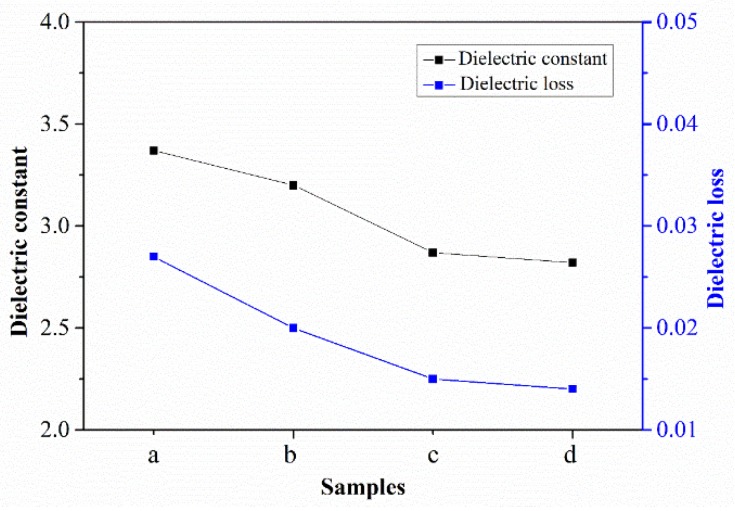
Dielectric properties of PEN–OH films.

What effect does the cross-linking reaction have on the dielectric properties of the material? Figure 9 shows the dielectric properties of PEN–OH films at 1 MHz with different heat-treatment temperatures, including dielectric constant and dielectric loss. It can be seen that sample a, without heat-treatment, shows a dielectric constant of 3.37 at 1 MHz. According to previous reports [13], the dielectric constant is 3.4, 4.1, 4.2, 4.5, 4.5 at 1 MHz in the PEN(BPA), PEN(PP), PEN(HQ/RS), PEN(PP/HQ), PEN(PPL), respectively. Compared to the above conventional PEN, this PEN–OH contains trifluoromethyl and shows a lower dielectric constant. As the heat-treatment temperature increased, the dielectric constant of the films decreased gradually. When the heat-treatment temperature reached 360 °C, the dielectric constant of sample d decreased to 2.82. The dielectric loss of the samples with different heat-treatment temperatures shows the same trend as the dielectric constant. The decrease of the dielectric constant and dielectric loss is mainly due to the fact that as the heat-treatment temperature increases, the degree of cross-linking reactions among the nitrile groups increases, resulting in the decrease of the amount of nitrile groups in the samples. The reduction of nitrile groups leads to the decline of the polarity of the material, resulting in a gradual decrease in the dielectric constant and dielectric loss of the samples.

The presence of nitrile groups is advantageous for the adhesion of the resin to the copper foil, while it is disadvantageous for lowering the dielectric constant. In other words, the occurrence of cross-linking reaction is unfavorable for adhesion, but advantageous for lowering the dielectric constant. Besides, the hot-press temperature has an effect on the fluidity of the PEN–OH, which will also affect the adhesion between the copper foil and the resin. Table 1 lists the 180° peel strength of the samples hot-pressed at different temperatures. It can be seen that the sample hot-pressed at 320 °C shows the best 180° peel strength of 1.01 N/mm. Compared to the sample hot-pressed at 300 °C, the sample hot-pressed at 320 °C possesses better liquidity, which is beneficial for the adhesion of the resin to the copper foil. When the hot-press temperature reaches 340 °C, the degree of cross-linking is too large, resulting in poor toughness of the material, and the decrease in nitrile group also leads to a decrease in adhesion to the copper foil. Therefore, the 180° peel strength of the sample hot-pressed at 340 °C decreased to 0.50 N/mm. It draws a conclusion that the FCCL only hot-pressed at the proper temperature possessed excellent peel strength.

## 4. Conclusions

A novel poly(arylene ether nitrile) terminated with hydroxyl groups (PEN–OH) was synthesized successfully, and the effects of heat-treatment temperature on the thermal properties, mechanical properties, and dielectric properties of the PEN–OH films have been studied in detail. The DSC and TGA analysis indicated that the *T*_g_ increased from 180.6 °C to 203.6 °C and the *T*_5%_ increased from 504.3 °C to 519.2 °C, with the heat-treatment temperature increasing from 200 °C to 360 °C. Moreover, as the heat-treatment temperature increased, the dielectric constant decreased from 3.4 to 2.8 at 1 MHz. Mechanical measurement showed the sample treated at 340 °C possessed the best tensile strength of 88.2 MPa and an elastic modulus of 2.3 GPa. Moreover, because of the excellent adhesive property of PEN–OH to copper foil, a double-layer FCCL without any adhesives based on PEN–OH was prepared by a simple hot-press method, and the effect of hot-press temperature on the peel strength of the FCCL was studied. The result shows that the FCCL hot-pressed at 320 °C possessed the highest peel strength with 1.01 N/mm. Therefore, the PEN–OH has potential applications in the electronic field.

## Figures and Tables

**Table 1:** Peel strength of the FCCL prepared at different hot-press temperatures.

Samples	Treated at 300 °C	Treated at 320 °C	Treated at 340 °C
180° peel strength(N/mm)	0.87	1.01	0.50
